# Transformer-Based Model with Dynamic Attention Pyramid Head for Semantic Segmentation of VHR Remote Sensing Imagery

**DOI:** 10.3390/e24111619

**Published:** 2022-11-06

**Authors:** Yufen Xu, Shangbo Zhou, Yuhui Huang

**Affiliations:** College of Computer Science, Chongqing University, Chongqing 400044, China

**Keywords:** swin transformer, remote sensing, semantic segmentation, dynamic attention pyramid head

## Abstract

Convolutional neural networks have long dominated semantic segmentation of very-high-resolution (VHR) remote sensing (RS) images. However, restricted by the fixed receptive field of convolution operation, convolution-based models cannot directly obtain contextual information. Meanwhile, Swin Transformer possesses great potential in modeling long-range dependencies. Nevertheless, Swin Transformer breaks images into patches that are single-dimension sequences without considering the position loss problem inside patches. Therefore, Inspired by Swin Transformer and Unet, we propose SUD-Net (Swin transformer-based Unet-like with Dynamic attention pyramid head Network), a new U-shaped architecture composed of Swin Transformer blocks and convolution layers simultaneously through a dual encoder and an upsampling decoder with a Dynamic Attention Pyramid Head (DAPH) attached to the backbone. First, we propose a dual encoder structure combining Swin Transformer blocks and reslayers in reverse order to complement global semantics with detailed representations. Second, aiming at the spatial loss problem inside each patch, we design a Multi-Path Fusion Model (MPFM) with specially devised Patch Attention (PA) to encode position information of patches and adaptively fuse features of different scales through attention mechanisms. Third, a Dynamic Attention Pyramid Head is constructed with deformable convolution to dynamically aggregate effective and important semantic information. SUD-Net achieves exceptional results on ISPRS Potsdam and Vaihingen datasets with 92.51%mF1, 86.4%mIoU, 92.98%OA, 89.49%mF1, 81.26%mIoU, and 90.95%OA, respectively.

## 1. Introduction

Propelled by the rapid development of remote sensing and sensor technology, large amounts of very-high-resolution remote sensing data have been obtained, which are still growing considerably. Delving into these fine-resolution remote sensing images, which contain rich spatial information and detailed features, is of great importance. Semantic segmentation, i.e., pixel-wise classification, is a fundamental task in exploiting RS images, which has received widespread attention. The essential goal of semantic segmentation is to identify the semantic category of every pixel in the RS image. However, enormous challenges reside in the complex background information, high resolution, various spectral information, and target structure variation. Currently, RS image semantic segmentation has been utilized in many real-world applications, such as urban planning [1], agricultural production [2], environmental protection [3], natural disaster damage assessment [4], mineral mining [5], marine exploration [6], and building extraction [7].

In recent years, deep learning, especially convolutional neural network, has been the mainstream method for semantic segmentation of remotely sensed images [8,9,10]. Compared with traditional segmentation methods based on machine learning, such as support vector machine [11] and random forest [12], CNN-based methods can capture more fine-grained local information. The Fully Convolutional Network (FCN) [13] is the ground-breaking network to effectively achieve satisfying segmentation results in an end-to-end manner. FCN was completely composed of convolution layers, replacing the original fully connected layers. However, the segmentation results were restricted by the over-simplified design of the decoder, resulting in coarse-resolution segmentation. Consequently, U-Net [14] was proposed with two symmetric branches of equal complexity and elegance, which consists of an encoder-named contracting path for extracting hierarchical features and a decoder-named expanding path for restoring spatial resolution. Subsequently, the encoder-decoder framework has established its status as the standard architecture for semantic segmentation of RS images by exhibiting exceptional results. However, due to the locality of the convolution operation, it is genuinely challenging to acquire a global context without increasing the network’s depth to gain a larger perceptive field. To solve this problem, existing literature intends to apply multi-scale fusion strategies to convolutional neural networks.

PSPNet [15] aggregated different-region-based based context through the Pyramid Pooling Module (PPM) while Deeplabv3 [16] augmented the Atrous Spatial Pyramid Pooling module (ASPP), both for the purpose of multi-scale context acquisition. DeeplabV3+ [17] followed the encoder-decoder architecture by adding an effective decoder module based on DeeplabV3 and applied depthwise separable convolution [18] to the Atrous Spatial Pyramid Pooling module. Zhang et al. [19] also adopted an encoder-decoder framework using the strip pool method to segment farmland vacancy from RS images. UperNet [20] further exploited the Pyramid Pooling Module to obtain a global context by utilizing features of different scales and achieved unified scene parsing. Liu et al. [21] proposed an end-to-end self-cascaded network which aggregated multi-scale contexts captured on the output of a CNN encoder in a self-cascaded manner. In addition, the attention mechanism is also a popular option for capturing contextual dependencies. DA-Net [22] designed parallel channel attention and position attention for the purpose of rich global information. Li et al. [23] introduced a cascaded residual attention mechanism to enhance road extraction from RS images. Nevertheless, instead of encoding global context directly, the aforementioned methods accumulated contextual information from local features acquired by convolution layers. As a consequence, obtaining accurate contextual information from RS images is still in demand.

Meanwhile, transformer-based models have demonstrated great potential in modeling long-range dependencies, which makes it easier to gain clear global information. DETR [24] proposed an end-to-end framework by combining a common convolutional neural network with transformer architecture. DETR took advantage of the global modeling capabilities of the transformer to handle object detection as a set prediction problem via bipartite matching. Vision transformer [25] directly applied the transformer in natural language processing to computer vision without considering the innate characteristics of visual signals. Correspondingly, vision transformer is only applicable for image classification tasks. Therefore, to address the problems of distinct scale variations of targets and high resolution of pixels in images, Swin Transformer [26] was proposed in a hierarchical architecture to capture features of different scales, along with the Shifted Window Multi-head Self-Attention (SW-MSA) mechanism to model globally. Therefore, Swin Transformer became suitable for many downstream vision tasks, such as object detection and semantic segmentation. Sun et al. [27] proposed HMRT semantic extraction network for remote sensing images by obtaining a global receptive field using transformer encoding and decoding. Wang et al. [28] introduced a bilateral awareness network which constituted a dependency path and a texture path by combining Transformer and Convolution to fully obtain long-range relationships and fine-grained details.

However, breaking images into patches to calculate attention ignores the intrinsic spatial information inside patches as each patch is compressed into a 1-D sequence. Furthermore, with only the encoder stage of the Swin Transformer, the detailed spatial resolution cannot be restored. Therefore, we propose a transformer-based encoder-decoder architecture adopting an Unet-liked shape called SUD-Net for RS images. SUD-Net constitutes a new dual encoder with Swin Transformer blocks and reslayers in reverse order to complement contextual features with fine-grained details through layers of different hierarchical semantic features. In addition, by adding a decoding path also composed of Swin Transformer blocks with upsampling layers in between, SUD-Net is capable of recovering sharper edges and achieving remarkable results. Furthermore, we designed a Multi-Path Fusion Module (MPFM) with Patch Attention (PA) to encode spatial information of patches and fuse features of different scales between transformer layers and reslayers effectively. Finally, we devised a Dynamic Attention Pyramid Head (DAPH) to attach to the end of SUD-Net, which could refine the feature maps and aggregate contextual and local information flexibly to better serve segmentation. In summary, our main contributions are as follows:

1. A new dual framework based on Swin Transformer Block and reslayers was constructed in reverse order. By obtaining coarse-grained resolution and fine-grained resolution simultaneously, SUD-Net is capable of gathering global context and detailed information effectively. Additionally, by adding a decoder composed of Swin Transformer blocks to upsample feature maps extracted by the encoder, SUD-Net can restore sharper edge maps and achieve satisfying segmentation results.
2. A Multi-Path Fusion Module is proposed between the reversed reslayers and transformer layers to adaptively fusion features containing different semantics. Patch attention was incoporated into MPFM to retrieve spatial information loss inside each patch and further fuse position information.
3. A Dynamic Attention Pyramid Head was designed to aggregate contextual and local information effectively and refine feature maps obtained by the backbone, which can further decode necessary high-level representations for segmentation.
4. SUD-Net achieves state-of-the-art results on the Potsdam dataset and comparatively satisfying results on the Vaihingen dataset of 92.51%mF1, 86.4%mIoU, 92.98%OA, 89.49%mF1, 81.26%mIoU, and 90.95%OA, respectively.

## 2. Methods

### 2.1. Architecture

**Figure 1:**
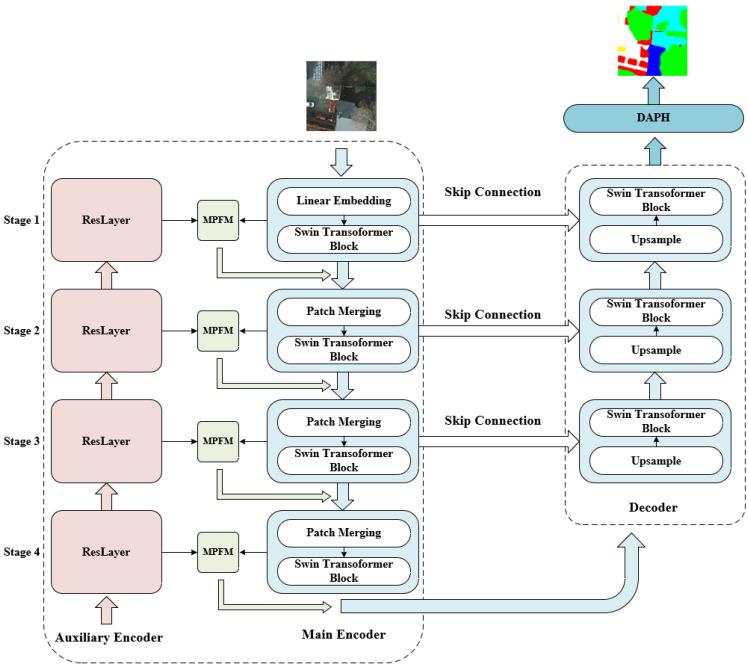
The overall architecture of SUD-Net.

Since transformer-based models can acquire long-range dependencies and convolutional neural networks can capture fine-grained local features, existing literature tends to construct U-shaped architecture based on transformer blocks and convolutional neural networks, which exhibit promising results on remote sensing datasets [29,30,31,32]. Inspired by these, we propose a novel dual encoder of two branches: Swin Transformer blocks and reslayers in reverse order, along with a decoder of only Swin Transformer blocks. The overall architecture of our proposed SUD-Net is illustrated in Figure 1.

The encoder of SUD-Net consisted of two paths: the main encoder and the auxiliary encoder. As for the auxiliary encoder, we used reslayers from ResNet34 [33] for its capability of capturing local detailed feature representation. Specifically, the reslayer and Swin Transformer block were fused by Multi-Path Fusion Module in reverse order. Therefore, by reshaping the output feature maps of 4 stages from ResNet34, the feature representation capability of our main encoder was enhanced and complemented by reslayers because the fused layers had disparate semantic information. ResNet was widely adopted in constructing deep neural networks as the backbone for various visual tasks, such as image classification, object detection, semantic segmentation, and instance segmentation. ResNet introduced residual connection and identity mapping to solve the problem of degradation problem as the networks get deeper. Finally, the Swin Transformer block was the basic component in the recently proposed Swin Transformer.

For a given RS image $X\in R^{H\times W\times 3}$, SUD-Net fed it into both encoders, which had 4 feature extraction stages. For the main encoder, *X* was split into non-overlapping patches with a dimension of 4×4×3=48 by Patch Partition. Following Patch Partition, we applied a linear embedding layer to project the value of $\frac{H}{4}\times \frac{W}{4}\times 48$ to $\frac{H}{4}\times \frac{W}{4}\times C_{1}$. The Swin Transformer block would maintain the shape of feature maps. So in order to obtain hierarchical feature maps, Patch Merging layers were designed to reduce the number of tokens and double the channels. As a result, Patch Merging layers would downsample the resolution fourfold. As for the decoder, we constructed a restoring path using Swin Transformer blocks and upsampling layers. The proposed upsampling layers had the opposite effect of expanding feature maps compared to Patch Merging. The output of auxiliary encoder stages is defined as $AE_{i}$, the main encoder is defined as $ME_{i}$, where i=1,2,3,4, and the decoder is defined as $D_{i}$, where i=1,2,3. The shape of $AE_{i}$ is $\frac{H}{2^{i+1}}\times \frac{W}{2^{i+1}}\times C_{2}2^{i-1}$, the shape of $ME_{i}$ is $\frac{H}{2^{i+1}}\times \frac{W}{2^{i+1}}\times C_{1}2^{i-1}$, and the shape of $D_{i}$ is $\frac{H}{2^{5-i}}\times \frac{W}{2^{5-i}}\times C_{1}2^{3-i}$. Since we intended to complement the output feature maps of Swin Transformer blocks with ResNet in reverse order, we needed to reshape $AE_{i}$ to $\frac{H}{2^{i+1}}\times \frac{W}{2^{i+1}}\times C_{1}2^{i-1}$ to match $ME_{i}$. In particular, we devised a Multi-Path Fusion Module to fuse $AE_{i}$ and $ME_{i}$ along with Patch Attention specifically designed for spatial information loss inside patches instead of the initial element-wise addition. Furthermore, SUD-Net adopts skip connections to concatenate the encoder and decoder features before reducing the channels using Bottleneck, which can be summarized as:

(1) $$f_{sc}\left(ME_{i+1},AE_{i+1},D_{i}\right)=B_{i}\left(Concat\left(f_{mpfm,i}\left(ME_{i+1},R_{i}\left(AE_{4-i}\right)\right),D_{i}\right)\right)$$

where i=1,2,3, $R_{i}$ denotes Reshape operation explicitly described in Equation (6), $f_{mpfm,i}$ represents Multi-Path Fusion Module expressed in Equation (9), Concat denotes Concatenation operation over channel dimension, and $B_{i}$ is a Bottleneck block composed of 1×1 convolution, Batch Normalization (BN) [34], and ReLU to halve the corresponding channels of stacked feature maps. In the end, SUD-Net applied a Dynamic Attention Pyramid Head to refine and aggregate feature maps adaptively to perform segmentation, producing the final segmented map.

**Figure 2:**
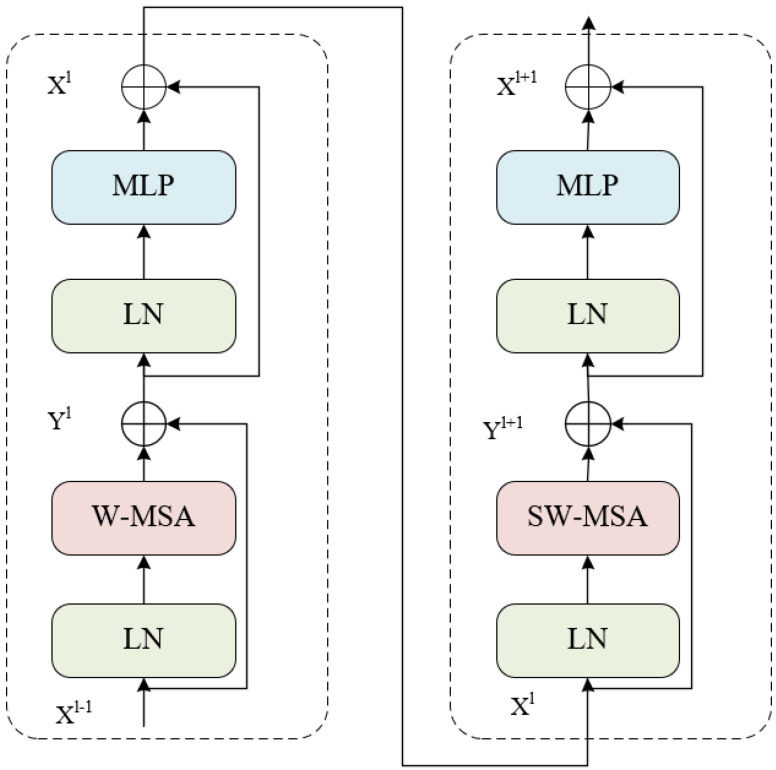
Two successive Swin Transformer Blocks.

Since the blocks in the standard Transformer [35] and Vision Transformer perform global Multi-Head Self-Attention (MSA), the computational complexity grows quadratically with respect to the number of tokens, causing great challenges in dense prediction tasks where there are substantial tokens. As a consequence, Swin Transformer blocks adopt a Window-Based Multi-Head Self-Attention (W-MSA) strategy and the computational complexity becomes linear concerning the image size. However, if MSA is only computed in non-overlapping windows, transformer architecture would no longer hold the ability to model long-range dependencies. Subsequently, Swin Transformer blocks apply cross window connection by shifting the window towards the bottom right direction by two patches, which is called Shifted Window-Based Multi-Head Self-Attention (SW-MSA). In this way, in the Swin Transformer blocks of later stages would be capable of perceiving a large portion of the image. As shown in Figure 2, a Swin Transformer stage is composed of two successive blocks: the first one performs W-MSA and the second one performs SW-MSA.

The computation details of Swin Transformer block are summarized as:

(2) $$Y^{l}=W-MSA\left(LN\left(X^{l-1}\right)\right)+X^{l-1}$$

(3) $$X^{l}=MLP\left(LN\left(Y^{l}\right)\right)+Y^{l}$$

(4) $$Y^{l+1}=SW-MSA\left(LN\left(X^{l}\right)\right)+X^{l}$$

(5) $$X^{l+1}=MLP\left(LN\left(Y^{l+1}\right)\right)+Y^{l+1}$$

where $X^{l}$ represents the output feature embedding of W-MSA, and $X^{l+1}$ denotes the output feature embedding of SW-MSA.

### 2.2. Multi-Path Fusion Module

**Figure 3:**
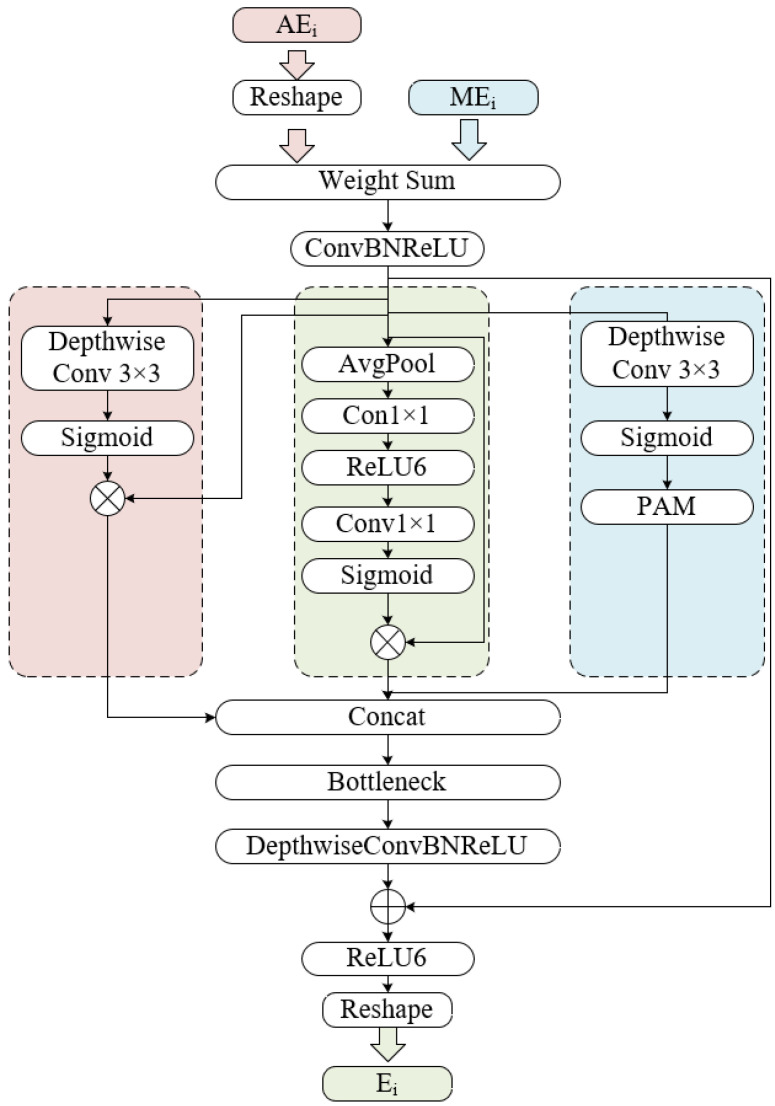
Multi-Path Fusion Model.

In order to efficiently complement the contextual information obtained by our main encoder with fine-grained local details extracted by our auxiliary encoder, we devised a Multi-Path Fusion Module with Patch Attention (PA), which can encode position information of different patches. The detailed implementation of MPFM is shown in Figure 3.

First, given the input as $AE_{i},ME_{i}\in R^{H\times W\times C}$, as the reversed feature maps extracted by reslayers from ResNet34 have a different shape with respect to corresponding Swin layers, areshape operation is conducted on the output layers from 4 stages of AE. In our default settings, the first two layers, i.e., $AE_{1},AE_{2}$, needed to be respectively compressed to $\frac{1}{4}$ and $\frac{1}{2}$ of its original resolution while channels were increased to $8C_{1}$ and $4C_{1}$ from $C_{2}$ and $2C_{2}$ correspondingly through a ConvBNReLU block. The last two layers, i.e., $AE_{3},AE_{4}$, were expanded to 4 times and 2 times its original resolution, respectively, and the channels were reduced to $C_{1}$ and $2C_{1}$ from $8C_{2}$ and $4C_{2}$ accordingly using a TransBNReLU block. The equation of our Reshape operation is presented as:

(6) $$f_{R,i}\left(AE_{i}\right)=\left\{\begin{array}{cc} \; & ReLU(BN\left(Conv\left(AE_{i}\right)\right))\;\mathrm{if}\;i=1,2\\ \; & ReLU(BN\left(Trans\left(AE_{i}\right)\right))\;\mathrm{if}\;i=3,4 \end{array}\right.$$

where Trans means ConvTransposed2D operation.

**Figure 4:**
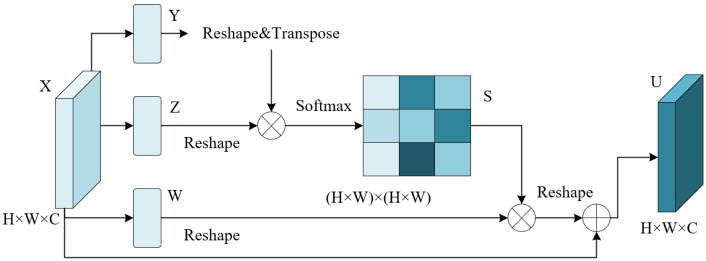
Position Attention Module.

Second, a weighted summation between reshaped $AE_{i}$ and $ME_{i}$ was performed, followed by a ConvBNReLU block before three paths of attention mechanisms. As for the left path, inspired by [36], we adopted Spatial Attention (SA), which was carried out by depthwise convolution to generate a spatial-wise attention feature map. With respect to the middle path, Channel Attention (CA) is applied through a collection of global average pooling operation, whose aim is to produce a channel attention map, 1×1 convolution for decreasing channels, ReLU6, 1×1 convolution for increasing channels to its original dimension and sigmoid activation function. Both attention paths were followed by matrix multiplication operation. With regard to the right path, Patch Attention (PA) was applied over depthwise convolution and the sigmoid activation function to obtain spatial patch maps. Then, in order to acquire the position information inside patches, the Position Attention Module (PAM) was conducted to introduce the patch position relationships over local features by taking full advantage of reslayers back to the network. The detailed structure of PAM is illustrated in Figure 4. Motivated by [22], PAM first produced a spatial matrix and performed matrix multiplication between the original matrix and the attention matrix. Then an element-wise matrix sum operation on the multiplied result and the original maps was performed to acquire the eventual representations. Given the fused layers as $X\in R^{H\times W\times C}$, convolution layers were used to produce two feature maps $Y\in R^{H\times W\times C}$, $Z\in R^{H\times W\times C}$, and $W\in R^{H\times W\times C}$, which were later reshaped to $R^{N\times C}$, where N=H×W. A matrix multiplication was then conducted between *Y* and $C^{T}$, followed by a softmax layer to gain $S\in R^{N\times N}$:

(7) $$s_{ji}=\frac{exp(Y_{i}\cdot Z_{j}^{T})}{\sum_{i=1}^{N}exp\left(Y_{i}\cdot Z_{j}^{T}\right)}$$

where $s_{ji}$ represents *i*th impact on *j*th position. We then performed a matrix multiplication between *W* and $S^{T}$ and reshaped the result to $R^{H\times W\times C}$. In the end, the result was multiplied by a scale parameter α, which was followed by an element-wise summation with *X*, leading to the final output $U\in R^{H\times W\times C}$. The Equation for *U* is as follows:

(8) $$U_{j}=\alpha \sum_{N}^{i=1}\left(s_{ji}^{T}W_{i}\right)+X_{j}$$

where the default value for α is 0 and can continuously learn to assign more weight to $s_{ji}^{T}W_{i}$ [37]. Therefore, the aforementioned modules can be formulated as:

(9) $$f_{mpfm,i}\left(ME_{i},AE_{i}\right)=Concat\left(SA\left(\alpha ME_{i}+\beta R_{i}\left(AE_{4-i}\right)\right),CA\left(\alpha ME_{i}+\beta R_{i}\left(AE_{4-i}\right)\right),PA\left(\alpha ME_{i}+\beta R_{i}\left(AE_{4-i}\right)\right)\right)$$

where i=1,2,3,4, α, and β denote adaptive weight assigned for $ME_{i}$ and $AE_{i}$, respectively, and Concat represents concatenation operation over channel dimension.

Third, all feature maps obtained from three attention paths were concatenated together over the channel dimension, which caused the channels of output maps to triple. Therefore, we designed a Bottleneck, the same as $B_{i}$ in Equation (1), to reduce the channel dimension to its original size, followed by a DepthwiseConvBNReLU block. To avoid network degeneration, a residual connection was added to the aforementioned module.

Finally, a reshape module composed of 3×3 convolution, BN, and ReLU was introduced to recover the channel to its corresponding input channel, and the output is denoted as $E_{i}$.

### 2.3. Dynamic Attention Pyramid Head

**Figure 5:**
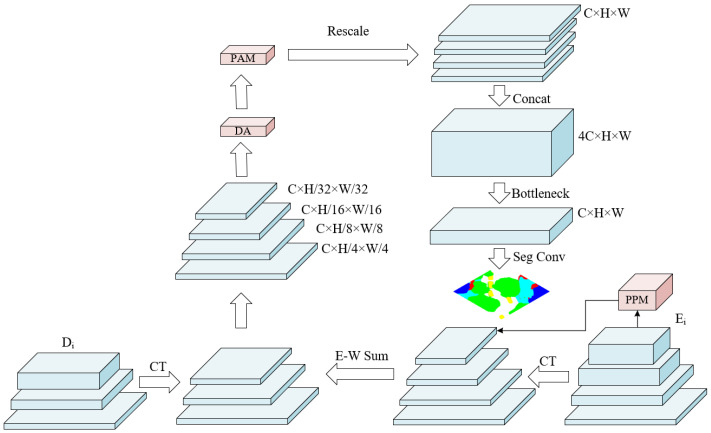
Dynamic Attention Pyramid Head.

Dynamic Attention Pyramid Head is introduced to further aggregate flexible spatial contextual and local information from feature maps obtained by both paths simultaneously. Inheriting the pyramid pooling design from PSPNet and adopting feature pyramid network structure, DAPH exhibits excellent segmentation performance attaching to the end of our backbone, whose structure is illustrated in Figure 5. Given the output of our encoder as $E_{i}$, where i=1,2,3,4, the output of our decoder is $D_{i}$, where i=1,2,3. Since $E_{i}$ and $D_{i}$ represent the corresponding output feature maps from the bottom up, according to Figure 5, the channels of $E_{i}$ and $D_{i}$ can be denoted as $C_{1}i$. In our default setting, $C_{1}=96$, which may change in our ablation study. First, we used a Channel Transformation (CT) module to change the channels of all feature maps to C=512. Second, an Element-Wise Sum (E-W Sum) operation was performed on $E_{i}$ and $D_{i}$, correspondingly, after imposing a Pyramid Pooling Module (PPM) from PSPNet on the last stage of our encoder, i.e., $E_{4}$. Third, a Dynamic Attention Module was specifically devised to adaptively focus on effective contextual and regional information, followed by a PAM. In addition, we rescaled the result feature maps ($\frac{H}{2^{i+1}}\times \frac{W}{2^{i+i}}\times C$) to the shape of H/4×W/4×C, where i=1,2,3,4. Furthermore, a Concatenation operation was performed over the channel dimension, which was followed by a Bottleneck block to reduce the channel back to *C*. Finally, the segmentation map of RS images was obtained through a simple 1×1 convolution layer.

DAPH fully utilizes a top-down architecture with lateral connections from both encoder and decoder to fuse semantic information of all-level features. Ref. [38] raised the problem that the empirical receptive field of a deep convolutional neural network is relatively inadequate, although the theoretical receptive field is presumably large. However, by introducing the Swin Transformer block into deep neural networks, models can have the capability to grasp the whole image as the stages move deeper. In order to further aggregate global representations, a Pyramid Pooling Module is appended to the last output layer of the encoder. Enlightened by [39], we attached a dynamic attention module as a connecting neck before enlarging the resolution of result feature maps to adaptively concentrate on effective semantics. Given the feature pyramid obtained by element-wise addition operation as $P\in R^{H\times W\times C\times L}$, where *L* represents the level of obtained feature pyramid, DA applies scale-aware attention to dynamically fuse features from different levels to distil semantic significance:

(10) $$f\left(P\right)=max(0,min\left(1,\left(f\left(\frac{1}{HW\times C}\sum_{HW,C}^{}P\right)+1\right)/2\right))\cdot P$$

DA learns to concentrate on discriminative areas existing in spatial locations and affected by feature levels by further imposing spatial-aware attention, which involves two steps. First, DA adopts deformable convolution [40] to make the attention learning sparse. Second, features across all the levels of clustering at the same spatial regions are aggregated, which can be formulated as:

(11) $$f\left(P\right)=\frac{1}{L}\sum_{l=1}^{L}\sum_{k=1}^{K}w_{l,k}\cdot P\left(l;p_{k}+\Delta p_{k};c\right)\cdot \Delta m_{k}\cdot P$$

where *K* denotes the number of sparse sampling locations, $p_{k}+\Delta p_{k}$ represents a shifted location by the self-learned spatial offset $\Delta p_{k}$ to focus on a discriminative region, $\Delta m_{k}$ denotes a self-learned importance scalar at location $\Delta m_{k}$, and $\Delta p_{k}$ can be learned from the input feature from the median level of *P*.

## 3. Experiments and Results

### 3.1. Experimental Settings

#### 3.1.1. Datasets Description and Preparation

In our experiments, we utilized the Potsdam and Vaihingen datasets, which were extensively used in the semantic segmentation task of RS images to verify the effectiveness of our proposed model: SUD-Net. Potsdam and Vaihingen datasets are benchmark datasets of aerial remote sensing images, which are collected and released by the International Society for Photogrammetry and Remote Sensing (ISPRS).

1. Potsdam Dataset
  We employed the Potsdam dataset for the 2D Semantic Labeling Contest, which contains 38 patches of 6000×6000 pixels. The true Othophoto (TOP) generated from a TOP mosiac in channel composition of RBG was used for training and testing. The ids of training patches are: 2_10, 2_11, 2_12, 3_10, 3_11, 3_12, 4_10, 4_11, 4_12, 5_10, 5_11, 5_12, 6_7, 6_8, 6_9, 6_10, 6_11, 6_12, 7_7, 7_8, 7_9, 7_10, 7_11, 7_12, and the rest patches are used for testing: 2_13, 2_14, 3_13, 3_14, 4_13, 4_14, 4_15, 5_13, 5_14, 5_15, 6_13, 6_14, 6_15, 7_13. The Potsdam dataset involves six classes of Impervious Surface, Building, Low Vegetation, Tree, Car, and Clutter. Since each patch is too big to be fed into the network considering limited GPU memory, we followed the common principle of dividing patches into smaller images. In our paper, each patch was split into a resolution of 512×512 with a stride of 256 in our default setting. As a result, we had 3456 images for training and 2016 images for testing, whose sizes were all 512×512.
2. Vaihingen Dataset.
  We employed the Vaihingen dataset for the 2D Semantic Labeling Contest, which contains 33 high-resolution TOP image tiles of different sizes. Following the same division principle, we split each image into 512×512 with a stride of 256. There are also six categories, the same as Potsdam. In our experiments, the utilized ids for training were 1, 3, 5, 7, 11, 13, 15, 17, 21, 23, 26, 28, 30, 32, 34, 37, and the rest was for testing.

Following [41,42,43], the “Clutter” Category was ignored when quantitative evaluation was conducted on both datasets. As for data augmentation methods in the training stage, resize, random crop, random flip with a probability of 0.5, and normalized operations were adopted. Photometric distortion was also applied to an image sequentially, with a probability of 0.5. In the testing stage, a multi-scale augmentation strategy, including resize, random flip, and normalize, was adopted.

#### 3.1.2. Implementation Details

In our experimental environment, we used NVIDIA Geforce RTX 3090 GPU for hardware and Pytorch [44] framework for software. As for hyperparameter configuration, we set batch size = 8, initial learning rate = $3\times 10^{-4}$, and training iterations = 28 k. The AdamW [45] optimizer, which is a variant of Adam [46] with decoupled weight decay (0.01 in default setting) and polynomial decay strategy for learning rate with 1500 iterations for warmup was adopted. Each stage of SUD-Net consists of two successive Swin Transformer blocks, including the decoder and the size of input images, and was fixed 512×512 in our default setting. Following most studies on semantic segmentation, cross-entropy loss, which is appropriate for common segmentation scenarios. was employed to train the SUD-Net.

#### 3.1.3. Evaluation Metrics

Average F1 (mF1), Mean Intersection over Union (mIoU), and Overall Accuracy (OA) were employed to evaluate the performance of our proposed model: SUD-Net. The three evaluation metrics were calculated according to the Confusion Matrix. The accuracy of each class was represented by the F1 score, which was a combination metric of Precision and Recall. As for Overall Accuracy, it is the ratio of correctly predicted pixels to the total number of pixels. All the calculation formulas are listed as follows:

(12) $$Precision=\frac{TP}{TP+FP}$$

(13) $$Recall=\frac{TP}{TP+FN}$$

(14) $$F1=2\times \frac{Precision\times Recall}{Precision+Recall}$$

(15) $$IoU=\frac{TP}{TP+FP+FN}$$

(16) $$OA=\frac{TP}{TP+FP+TN+FN}$$

where TP represents true positive, FP represents false positive, TN represents true negative, and FN represents false negative. For a particular category, the F1 score is adopted to evaluate a model’s performance. mIoU and mF1 is computed as the mean value of IoU and F1 score among all categories, respectively.

### 3.2. Results

#### 3.2.1. Comparison of SUD-Net and Other Networks

Extensive experiments were conducted on ISPRS Potsdam and Vaihingen Datasets to compare the effectiveness of our proposed model and other state-of-the-art methods. Comparison of different models on Potsdam Datasets was performed both quantitatively and qualitatively. Quantitative results are displayed in Table 1. We compared our proposed SUD-Net with ERFNet [47], PSPNet [15], Deeplabv3+ [16], UperNet [20], CCNet [48], STransFuse [49], and STUnet [30]. As indicated in Table 1, our proposed SUD-Net surpassed all other models, with remarkable results of 92.57%mF1, 86.4%mIoU, and 92.98%OA benefiting from the global context modeling capabilities and restoring the resolution quality through its unique and elegant architecture.

ERFNet [47] adopts FCN as the decoder head while the next four models all adopt ResNet50 as the backbone to extract multi-level features. As seen from Table 1, the aforementioned models mainly composed of convolutional layers are only able to achieve 91.52%mF1, 84.63%mIoU, and 92.44%OA for best results. This validates the problem raised by [38] that the receptive field of deep convolutional neural networks in practice is inadequate, which leads to incompetent segmentation results. STransfuse [49] combines Transformer blocks with CNN to model a global semantic relationship. Nevertheless, without a proper decoder to expand the resolution of feature maps, STransfuse would only achieve insufficient results of 82.08%mF1, 71.46%mIoU, and 86.71%OA. STUnet [30] constructs a dual encoder structure of Swin Transformer and CNN in parallel, leading to better performance compared with STransfuse. It is perfectly clear that SUD-Net attains the highest F1 score in all categories (Numbers in bold font indicate the best results with reference to the corresponding column). Compared to the previous models, SUD-Net outperforms them by 1.05% mF1, 1.77%mIoU, and 1.01%OA with regard to corresponding highest scores.

**Figure 6:**
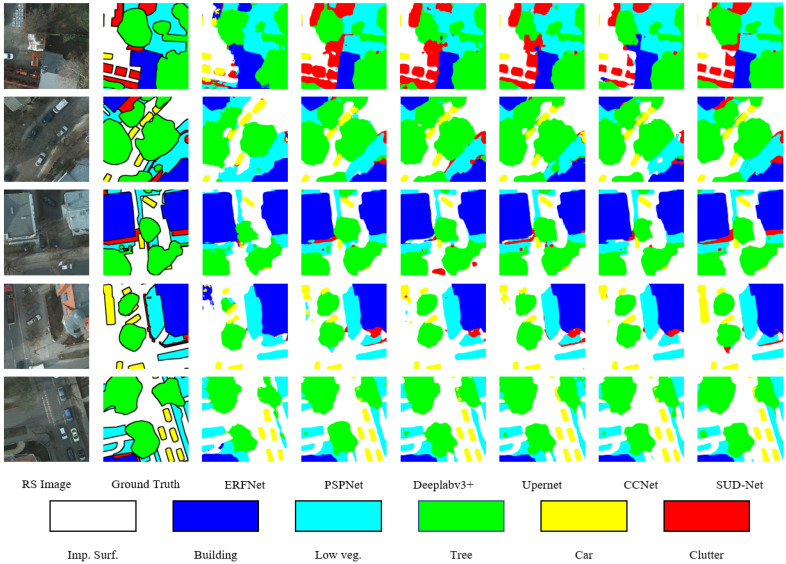
Visualization results of different models on five images randomly selected from testing set of Potsdam dataset.

In order to further demonstrate the capability of our proposed SUD-Net to capture important features in RS images, we compared the ability of different models to recognize different categories of ground objects. Visualization results of other networks on six randomly-selected images from Potsdam dataset for testing are shown in Figure 6. According to Figure 6, it is clear that our proposed model produced finer segmentation maps compared to previous methods. In the first row, ERFNet noticeably lacks the ability to model long-range dependencies, which mistakenly recognizes “Clutter” as “Car” for the first image. There are also several misclassifications in other areas. Furthermore, the output segmentation map of ERFNet exhibits a serious mosiac effect. PSPNet with a Pyramid Pooling Module is able to capture objects with different scales and Deeplabv3+ adopting dilated convolution, which leads to a larger receptive field that can achieve better visual segmentation results than ERFNet, as demonstrated in Figure 6. Upernet, obtaining global context information by utilizing feature pyramid network and Pyramid Pooling Module simultaneously, produces segmentation maps with sharper and clearer edges. CCNet with criss-cross attention acquires full-image dependencies in a more efficient way, leading to a minor increase of 0.18%mF1, 0.34%mIoU, and 0.14%OA compared to Upernet, which is inconspicuously indicated in the seventh column. Although Upernet succeeds in recognizing some indistinct clutter in the third and fourth row, CCNet decreases the probabilities of miscategorizing objects, such as the last row over the top right tree region, according to the ground truth. The aforementioned methods all encode contextual information in a mediate fashion or aggregate global contexts over local feature representation. In contrast, SUD-Net directly encodes global information using Transformer blocks and utilizes convolution layers simultaneously to obtain sufficient representations. As demonstrated in Figure 6, SUD-Net successfully categorizes most ground objects compared to previous methods.

#### 3.2.2. Ablation Studies

Comprehensive ablation experiments were conducted on both Potsdam and Vaihingen datasets, which include three aspects. According to our proposed architecture and modules, we carried out five submodels by gradually adding our proposed modules. Exhaustive experimental results are demonstrated in Table 2. The first one (a) denotes simply using Swin-T as the backbone and FCN as the decode head without altering any other modules or parameters for comparison. We constructed the second model (b) as our baseline by building a U-shaped network consisting of a Swin Transformer and convolutional layers simultaneously with FCN head, which yielded a performance gain of 1.13%mF1, 1.87%mIoU, and 0.18%OA respectively. Especially in the category “Car”, (b) dramatically increases its F1 score by 6.38%. As for (c), MPFM is incorporated into (b) to adaptively fuse features of different semantic information, bringing an increase of 0.36%mF1, 0.63%mIoU, and 0.14%OA. In addition, DAPH is integrated into (c) by replacing the FCN head, which can further improve the performance of our network by dynamically aggregating contextual and local representations (d). DAPH brings a 1.17%mF1, 1.09%mIoU, and 0.48%OA increase to the previous network. Finally, by incorporating all three modules into a complete network, SUD-Net (e) achieves state-of-the-art results on the Potsdam dataset. As for the effectiveness of our proposed model on Vaihingen dataset (Table 3), we will not further elaborate as the results are similar to Potsdam. Above results and analyses prove our proposed modules effective and efficient.

**Figure 7:**
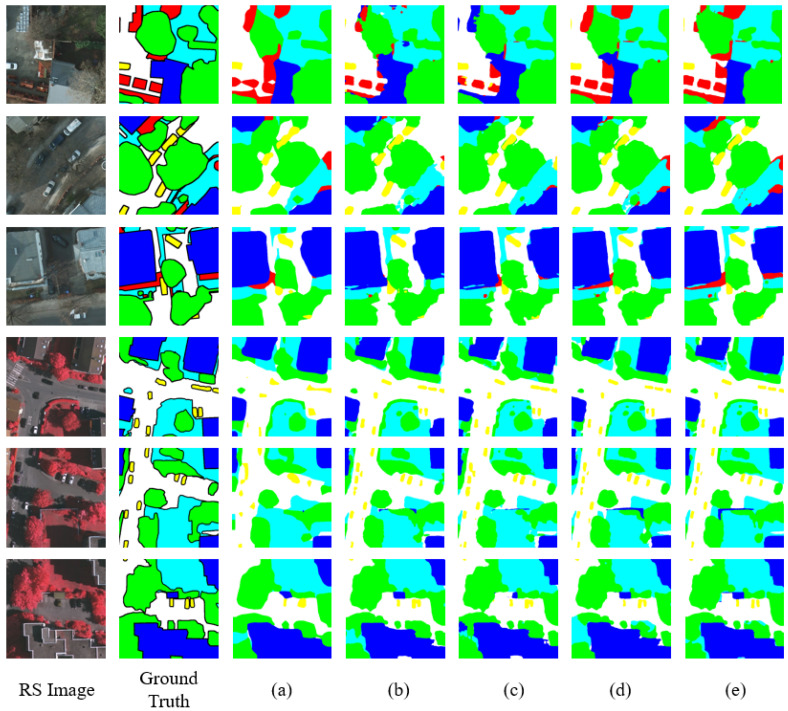
Visualization results of proposed modules on Potsdam and Vaihingen datasets. (**a**) FCN-head_Swin-t. (**b**) FCN-head_Swin-Res34-Unet. (**c**) FCN-head_Swin-Res34-Unet_Mpfm. (**d**) Daph_Swin-Res34-Unet. (**e**) Daph_Swin-Res34-Unet_Mpfm.

Visualization results on Potsdam and Vaihingen datasets of our proposed modules are shown in Figure 7. As illustrated in Figure 7, in the first row, after adopting Swin Transformer blocks, (b) can capture long-range dependencies by separating three blocks of clutter instead of attached together and the edges of objects become more clear and fine-grained. Applying MPFM to (b) and (c) can focus on detailed regions and eliminate falsely classified small objects. In addition, the DAPH-integrated model is capable of correctly classifying most ground objects after aggregating effective information using our proposed head. Especially on the top left, (d) fully distinguishes “Clutter” from “Tree”. In the last step of integrating all proposed architecture and modules, SUD-Net successfully categorizes all ground objects and produces a segmentation map with higher accuracy with a few disparities from ground truth. However, for the misclassified objects, for instance, the left region of the building, colored blue, is categorized as clutter due to the confusing roof with a complex surface. With regard to the red spot over the “Tree” region in the right bottom corner, there is actually clutter over the “Tree”, although with severe occlusion, whose color is evidently different from the “Tree” in the RS image. As a consequence, our model exhibits exceptional results according to above results and analyses. Since there exists some missed labels in the ground truth, SUD-Net hardly misclassifies objects according to the actual image.

As for the specific design of skip connections, we conducted experiments on two different skip connections: (a) Pixel-wise Addition (PA) and (b) Map-wise Concatenation (MC). In this experiment, reslayers-incorporated dual encoder-decoder architecture with an FCN head was adopted as our baseline. The results are indicated in Table 4, which demonstrates the effectiveness of map-wise concatenation.

Models tend to be influenced by the input resolution of RS images [29] considering that different input image sizes have various impacts on the final performance in Swin Transformer. Therefore, comparative experiments on the Potsdam dataset are based on our proposed U-shaped encoder-decoder architecture. By utilizing input sizes of 128×128, 256×256, and 512×512, models are trained and evaluated following the same experimental settings. As indicated in Table 5, increasing the input resolution of RS images results in performance gains of 3.4%mF1, 5.6%mIoU, and 3.35%OA, respectively, from 128×128 to 512×512. At the same time, the GFLOPs representing computation complexity is also increasing. Considering the rich spatial information contained in RS images, we chose to adopt a larger resolution 512×512 in our default setting in order to accomplish satisfying results.

Based on the original Swin Transformer, we also conducted ablation experiments about Swin variants on the Potsdam dataset. In this section, U-shaped encoder-decoder architecture was also applied to evaluate the impact of different model sizes. Following the configuration in Swin Transformer with small modifications specifically altered for U-shaped design, the detailed architecture specifications of Swin-Unet-T, Swin-Unet-S, and Swin-Unet-B are listed as follows:

- Swin-Unet-T: $C_{i}$ = 768, 384, 192, 96, $S_{i}$ = 2, 2, 2, 2, 2, 2, 2, $H_{i}$ = 3, 6, 12, 24, 24, 12, 6
- Swin-Unet-S: $C_{i}$ = 768, 384, 192, 96, $S_{i}$ = 2, 2, 18, 2, 2, 4, 2, $H_{i}$ = 3, 6, 12, 24, 24, 12, 6
- Swin-Unet-B: $C_{i}$ = 1024, 512, 256, 128, $S_{i}$ = 2, 2, 18, 2, 2, 4, 2, $H_{i}$ = 4, 8, 16, 32, 32, 16, 8

where $C_{i}$ denotes the channel dimension of the output feature pyramid acquired by the backbone, $S_{i}$ is the number of Swin Transformer blocks in each stage, and $S_{i}$ defines the number of heads computed within self-attention. The results of our ablation study concerning the Swin variants are demonstrated in Table 6. It is clear that by increasing the capacity of models, we can achieve better performance. However, the parameters of models also grow dramatically, which leads to more computational resources.

## 4. Conclusions

In this paper, we propose a novel dual branch encoder-decoder architecture consisting of Swin Transformer blocks and reslayers with a Dynamic Attention Pyramid Head called SUD-Net. Incorporating reslayers from Res34 into our encoder path in a reversed fashion complements the extracted global representations with fine-grained features. Targeted at the spatial loss problem inside patches, Multi-Path Fusion Module with Patch Attention was devised to recover position information and further fuse features of different scales adaptively. Furthermore, a Dynamic Attention Pyramid Head was constructed to append to the output of all stages from both the encoder and decoder. Experiments on ISPRS Potsdam and Vaihingen datasets verify the effectiveness of our proposed SUD-Net, which delivers satisfying segmentation results of 92.57%mF1, 86.4%mIoU, and 92.98%OA. Meanwhile, after observing the real RS images, ground truth may show a few missing or incorrect labels. However, SUD-Net still produces authentic and accurate segmentation maps according to visualization results. In the future, we will consider reducing the parameters of our proposed model and constructing a more lightweight model that can function at real-time speed. Furthermore, multi-modal data of RS images should also be taken into account to enhance segmentation performance.

## Figures and Tables

**Table 1:** Comparison of SUD-Net and other state-of-the-art networks on Potsdam dataset.

Model	Imp. surf.	Building	Low veg.	Tree	Car	mF1(%)	mIOU(%)	OA(%)
ERFNet [47]	88.38	92.38	80.02	78.34	87.62	85.35	74.82	87.08
PSPNet [15]	91.99	95.49	84.26	87.79	95.24	90.95	83.69	91.34
Deeplabv3+ [16]	91.21	95.43	85.46	87.47	94.47	90.81	83.39	90.86
Upernet [20]	92.27	95.89	86.17	87.48	94.88	91.34	84.29	91.63
CCNet [48]	92.15	96.02	85.39	88.4	95.64	91.52	84.63	91.97
STransFuse [49]	89.75	93.92	82.91	83.61	88.51	82.08	71.46	86.71
STUNet [30]	79.19	86.63	67.89	66.37	79.77	86.13	75.97	-
SUD-Net(Ours)	**93.61**	**96.98**	**87.63**	**88.7**	**95.95**	**92.57**	**86.4**	**92.98**

- means not reported in the original paper.

**Table 2:** Ablation results of different modules on Potsdam dataset.

Model	FCN-Head	Swin-Res34-Unet	MPFM	DAPH	Imp. surf.	Building	Low veg.	Tree	Car	mF1(%)	mIOU(%)	OA(%)
(a)	*√*				92.31	96.42	86.68	88.23	87.43	90.21	82.38	92.07
(b)	*√*	*√*			92.65	95.72	86.73	87.78	93.81	91.34	84.25	92.25
(c)	*√*	*√*	*√*		92.99	96.04	86.74	88.13	94.63	91.7	84.88	92.39
(d)		*√*		*√*	93.38	96.67	87.52	88.75	95.37	92.87	85.97	92.87
(e)		*√*	*√*	*√*	93.61	96.98	87.63	88.7	95.95	92.57	86.4	92.98

**Table 3:** Ablation results of different modules on Vaihingen dataset.

Model	FCN-Head	Swin-Res34-Unet	MPFM	DAPH	Imp. surf.	Building	Low veg.	Tree	Car	mF1(%)	mIOU(%)	OA(%)
(a)	*√*				90.7	94.99	82.28	88.41	67.18	84.71	74.63	89.56
(b)	*√*	*√*			91.99	95.27	81.63	88.32	84.77	88.4	79.55	90.06
(c)	*√*	*√*	*√*		92.14	95.25	82.98	88.78	84.18	88.67	79.95	90.42
(d)		*√*		*√*	92.29	95.55	83.21	89.09	86.35	89.3	80.94	90.73
(e)		*√*	*√*	*√*	92.89	95.73	83.51	88.96	86.36	89.49	81.26	90.95

**Table 4:** Ablation results of different skip connections on Potsdam dataset.

Skip Connections	mF1(%)	mIOU(%)	OA(%)
PA	91.11	84.07	92.09
MC	91.34	84.25	92.25

**Table 5:** Ablation results of input resolution on Potsdam dataset.

Image Size	mF1(%)	mIOU(%)	OA(%)	GFLOPs
128×128	87.94	78.65	88.9	9.55
256×256	90.33	82.55	91.22	37.08
512×512	91.34	84.25	92.25	143.68

**Table 6:** Ablation results of different Swin variants on Potsdam dataset.

Swin Variants	mF1(%)	mIOU(%)	OA(%)	Params(M)
Swin-Unet-T	91.34	84.25	92.25	74.68
Swin-Unet-S	92.55	86.35	93.16	106.66
Swin-Unet-B	92.57	86.38	93.09	165.70

## Data Availability

The ISPRS Potsdam and Vaihingen datasets used to support the results of this study are available online at https://www.isprs.org/education/benchmarks/UrbanSemLab/default.aspx (accessed on 1 November 2022).
